# Improved understanding of rice amylose biosynthesis from advanced starch structural characterization

**DOI:** 10.1186/s12284-015-0055-4

**Published:** 2015-06-17

**Authors:** Enpeng Li, Alex Chi Wu, Juan Li, Qiaoquan Liu, Robert G Gilbert

**Affiliations:** Tongji School of Pharmacy, Huazhong University of Science and Technology, Wuhan, Hubei 430030 China; Centre for Nutrition and Food Sciences, Queensland Alliance for Agricultural and Food Innovation, The University of Queensland, Brisbane, QLD 4072 Australia; Key Laboratory of Plant Functional Genomics of Ministry of Education, College of Agriculture, Yangzhou University, Yangzhou, Jiangsu 225009 China

**Keywords:** Fluorophore-assisted carbohydrate electrophoresis, Size exclusion chromatography, Chain length distribution, Modeling, Amylose biosynthesis, Starch branching enzyme, Molecular structural characterization

## Abstract

**Background:**

It has been shown from the chain length distributions (CLDs) that amylose chains can be divided into at least two groups: long and short amylose chains. These molecular structures influence some functional properties of starch, such as digestibility and mouth-feel. GBSSI is the key enzyme for the elongation of amylose chains; however, the effect of other starch biosynthesis enzymes in amylose synthesis is still not fully understood. Two advanced starch characterization techniques, size exclusion chromatography (SEC) and fluorophore-assissted carbohydrate electrophoresis (FACE), together with a newly developed starch biosynthesis model, are used to improve understanding of amylose biosynthesis.

**Results:**

SEC and FACE were used to determine the CLD of amylose and amylopectin in various native and mutant rice starches. The types of starch branching enzymes (SBEs) involved in the synthesis of the distinct features seen for shorter degrees of polymerization, DP, < 2000, and longer (DP > 2000) amylose chains are identified by combining these data with a mathematical model of amylopectin biosynthesis. The model enables each feature in the amylopectin CLD to be parameterized in terms of relative SBE activities, which are used to explain differences in the genotypes.

**Conclusions:**

The results suggest that while GBSSI is the predominant enzyme controlling the synthesis of longer amylose chains, some branching enzymes (such as BEI and BEIIb) also play important roles in the synthesis of shorter amylose chains.

## Background

Starch is a homopolymer of glucosyl units, with two main components: amylose and amylopectin. Amylose is a slightly branched molecule, and amylopectin is highly branched (Buleon et al. 1998; Tester et al. 2004). Amylose molecules usually have 3 to 11 branches, and the average length of the chains from debranched amylose molecules is degree of polymerization (DP) ~ 200 (Wang and White 1994; Yoshimoto et al. 2000). Amylopectin branches can be divided into three groups (A type, B type, and C type) depending on their organization in the amylopectin clusters. A type branches are the outer chains which carry no branches, and their average length is DP 6–16 (Hizukuri 1985, 1986). B type branches are the chains carrying branches; B type branches can be divided into B_1_, B_2_, B_3_, and B_4_ chains depending on number of clusters traversed (Wang and White 1994; Hizukuri 1986; Mua and Jackson 1997). Each amylopectin molecule only has one C type branch, which has the reducing end.

Granule-bound starch synthase I (GBSSI) is the key enzyme for amylose biosynthesis. Two amylose synthesis models have been proposed based on *in vitro* experiments. One model suggests that malto-oligosaccharides (MOSs) act as the primer for amylose chain synthesis, and without an MOS one has longer amylopectin chains rather than amylose chains (Ball et al. 1998). The other model suggests that amylose synthesis starts with a high molecular weight primer inside the starch granule (Ball et al. 1998).

Although it is well known that elongation of amylose chains is predominantly by GBSSI and that amylose branches are formed by starch branching enzymes (SBEs) (Denyer et al. 2001; Ball et al. 1998), little is known about the details of how the individual isoforms of SBE and starch synthase (SS) contribute to the amylose chain-length distribution (CLD). The lengths, and the relatively few but significant numbers, of these chains influence functional properties such as digestion rate and location, and mouth-feel (Syahariza et al. 2013). Previous studies have shown that debranched amylose distributions have at least two distinct groups (Ward et al. 2006; Syahariza et al. 2013; Li et al. 2011; Witt et al. 2012), which are here referred to as the short and long amylose features. Their synthesis may be controlled by different enzyme combinations.

Previous studies on amylose synthesis are mostly based on *in vitro* experiments; the present study uses advanced starch structural analysis and interpretation techniques to give a new insight into *in vivo* amylose synthesis. Fluorophore-assisted carbohydrate electrophoresis (FACE) and size exclusion chromatography (SEC) are used to analyze the CLDs of amylopectin and amylose molecules in wild-type and transgenic rices, the transgenic lines lacking one or more SBEs. A model (Wu and Gilbert 2010; Wu et al. 2013) for the amylopectin biosynthetic process is used to fit the amylopectin CLD obtained using FACE to incorporate the underlying mechanism (enzyme activity information) of amylopectin synthesis. This activity information will be seen to aid understanding of the amylose biosynthesis mechanism by seeing which activity change in amylopectin is reflected in a change in amylose CLD. This provides a means to examine which starch-synthesis enzymes besides GBSSI affect the synthesis of the different amylose components.

## Results

### CLDs of amylopectin

**Fig. 1:**
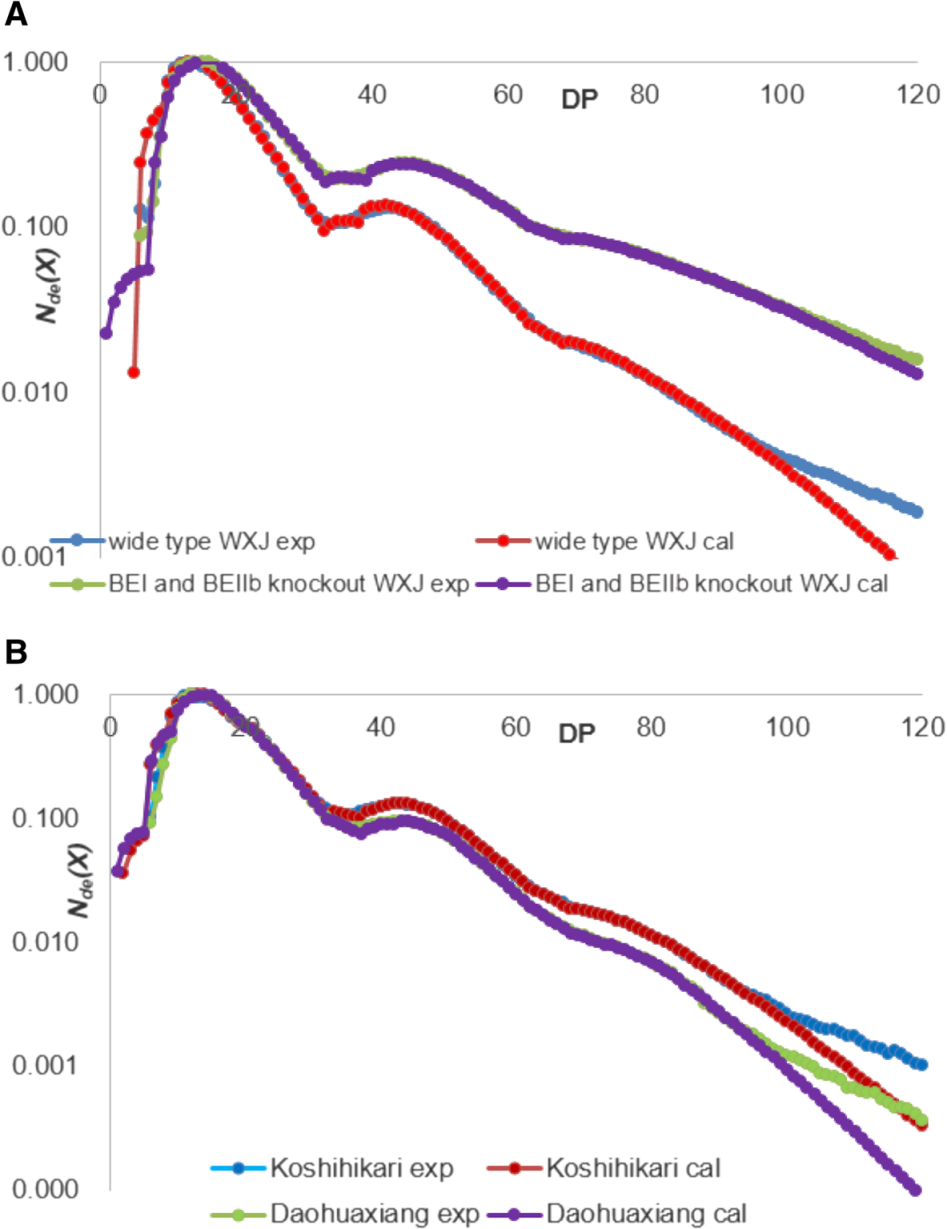
Experimental (“exp”, from FACE) and model-fitted (“cal”) number chain-length distributions (*N*_de_(*X*), arbitrary normalization) of debranched amylopectin (DP < 100) from wild type and BEI and BIIb knockout rice (**a**), from Koshihikari and Daohuaxiang (**b**)

The experimental and model-fitted CLDs of amylopectin from all rice samples obtained using FACE, normalized to their highest peaks, are shown in Fig. 1. The parameters in the model are given in the Methods section. BEI and BEIIb knockout WXJ rice amylopectin has relatively more long chains (DP > 15), as is reflected in larger *h*_(iii/i)_ and *h*_(v/i)_ values in the model fitting than those of the wild type WXJ rice (Table 1). The Wu-Gilbert model fitting result (Table 1) shows that the values of the most important parameters governing the shape of the amylopectin CLD, β_(ii)_, β_(iv)_ and β_(vi)_ are not very different between both rice starches, while those of β_(i)_, β_(iii)_ and β_(v)_ (and hence γ_(i,ii)_, γ_(iii,iv),_ γ_(v, vi)_) for wild-type WXJ rice starch are significantly larger than those of BEI- and BEIIb-knockout WXJ rice starch.

**Table 1:** Parameter values from fitting the biosynthesis model to the amylopectin CLDs of four different rice lines^a^

Samples	β_(i)_	β_(ii)_	β_(iii)_	β_(iv)_	β_(v)_	β_(vi)_	γ_(i,ii)_	γ_(iii,iv)_	γ_(v,vi)_	*h*_(iii/i)_	*h*_(v/i)_	*X*_AM1_	*X*_AM2_
Wild type	0.15	0.0115	0.0659	0.00934	0.0453	0.0000028	0.0582	0.0507	0.0361	0.113	0.00965	920	2172
BEI & BEIIb knockout	0.0831	0.0118	0.0468	0.00642	0.0298	0.0001	0.005	0.0355	0.027	0.202	0.0419	933	2141
Daohuaxiang	0.0921	0.0354	0.0527	0.0282	0.0465	0.012	0.0585	0.055	0.047	0.0823	0.00909	675	4711
Koshihikari	0.104	0.038	0.0485	0.0394	0.0461	0.0298	0.0584	0.0556	0.0489	0.115	0.00593	664	1899

^a^The explanation of all the parameters are included in the method section

Koshihikari rice amylopectin has relatively more long chains (DP > 30) than Daohuaxiang rice starch. Reflecting this, the values of β_(i)_, β_(ii)_, β_(iii),_ β_(v),_ γ_(i,ii)_, γ_(iii,iv) and_ γ_(v,vi)_ are similar between the two rice lines, and Koshihikari has higher β_(iv)_ and β_(vi)_ values than Daohuaxiang. The value of *h*_(iii/i)_ of Koshihikari is higher than that of Daohuaxiang, while *h*_(v/i)_ of Koshihikari is lower.

### CLDs of amylose

**Fig. 2:**
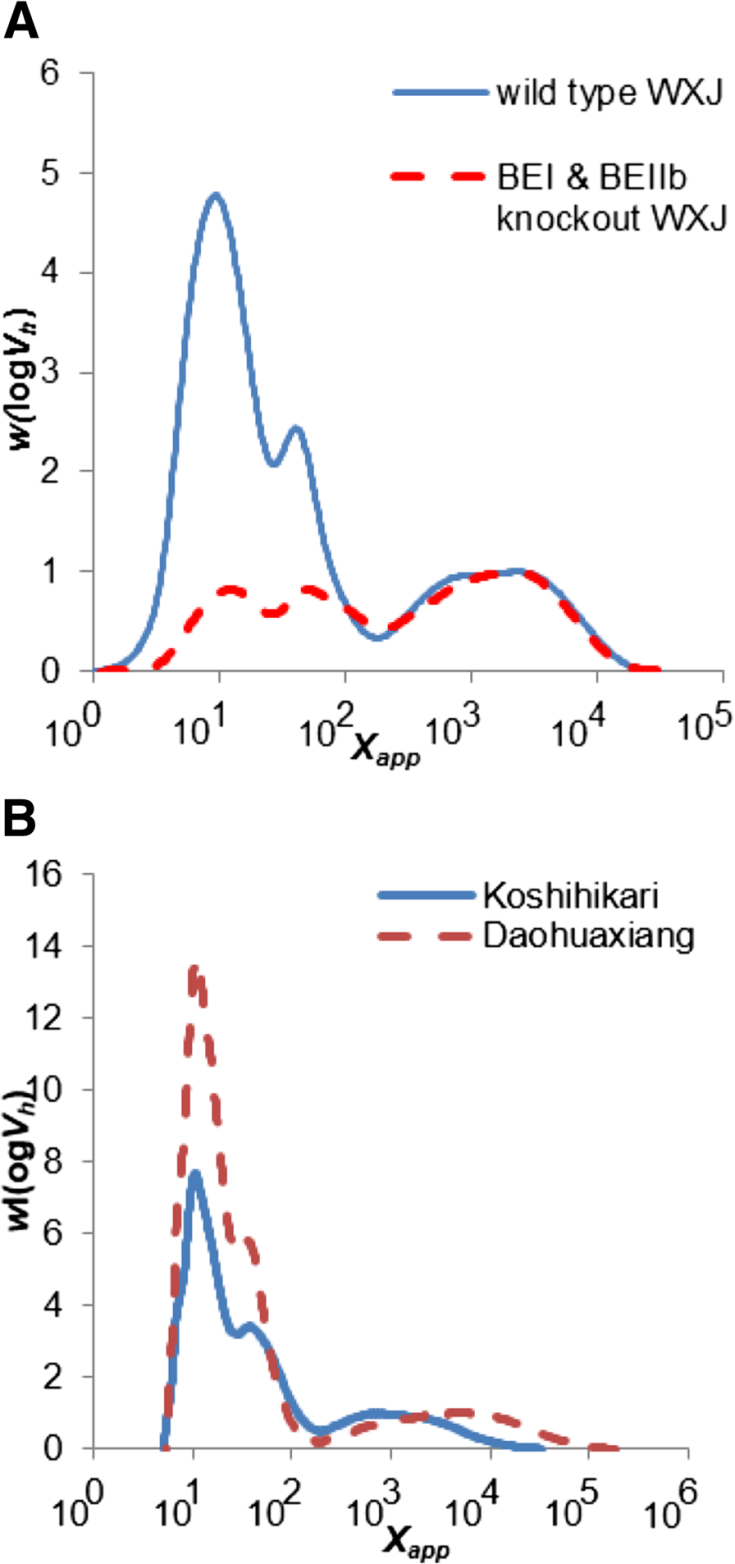
SEC weight chain-length distributions (arbitrary units) of debranched starch from wild type and BEI & BIIb knockout rice (**a**), from Koshihikari and Daohuaxiang (**b**)

**Fig. 3:**
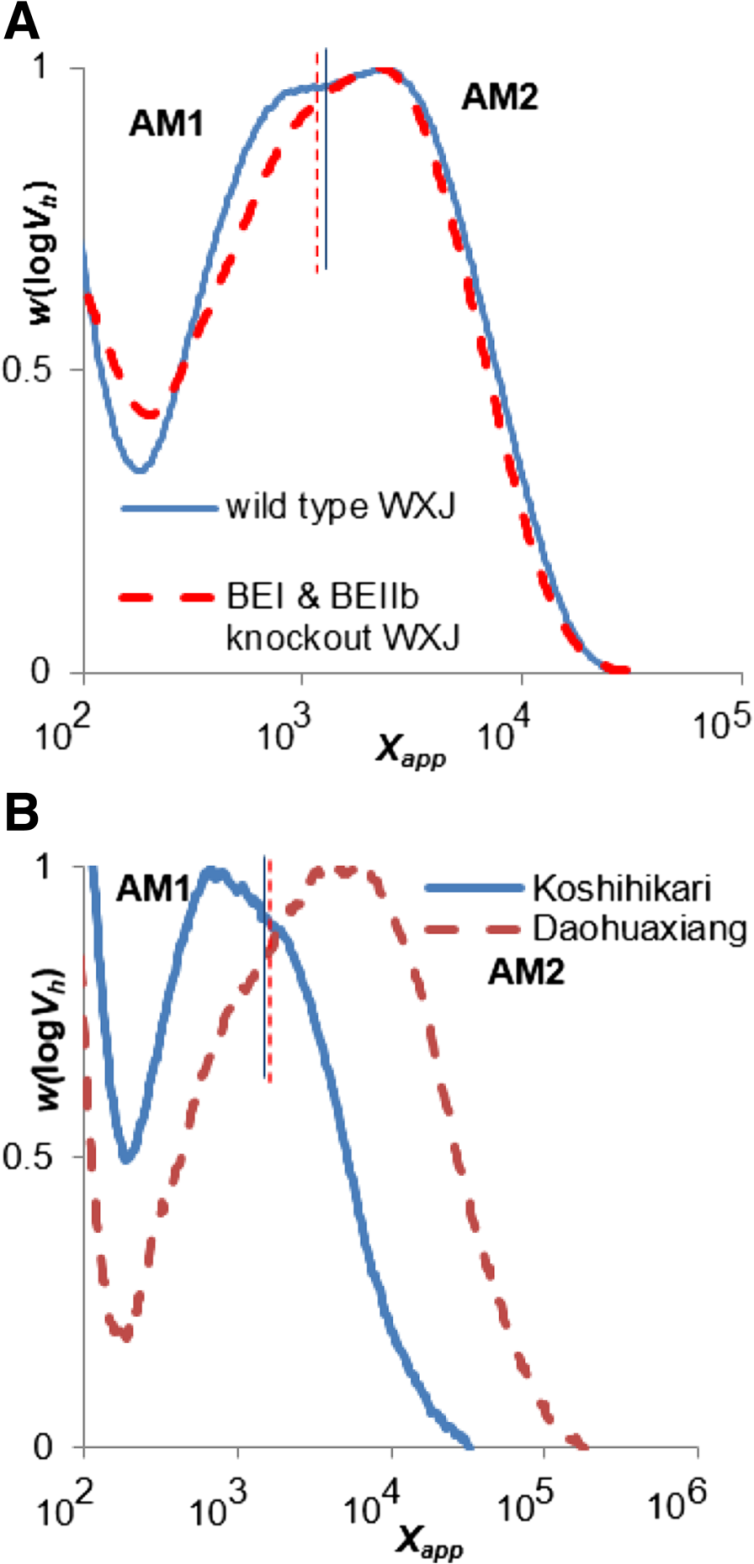
Data of Figure 2 with changed scale to show the amylose region, which is defined as those chains above the minimum in DP > 100. The limited calibration means that the DP axis is only semi-quantitative

Amylose CLDs were obtained by universal calibration from SEC analysis; because of the limited calibration range, the amylose results cannot be quantitatively compared to those from FACE in the DP region where these overlap. Amylose CLDs are reported here in terms of apparent DP *X*_app_, in cognizance of the limitations of the restricted calibration used here. SEC also suffers from band-broadening, which can mask subtle features (Castro et al. 2005). This section concerns only the amylose components, which can only be obtained from SEC, which, as just noted, suffers inaccuracies; those of amylopectin obtained from FACE, discussed in the preceding section, do not suffer from these inaccuracies, so the amylopectin component obtained from SEC is not discussed. The CLDs of amylose chains are normalized to their highest amylose peaks and are shown in Fig. 2 and Fig. 3. As seen elsewhere, *e.g.* (Ward et al. 2006; Syahariza et al. 2013; Li et al. 2011; Witt et al. 2012), if the SEC separation is sufficiently good, at least two distinguishable features are seen in rice amylose CLDs. We denote those seen here as the short-chain feature AM1, ranging from DP 150 to 2000, and the long-chain feature AM2, ranging from DP 2000 to 10000. *X*_AM1_ and *X*_AM2_ refer to the corresponding peak positions. The ratio of the AM1 area under peak (AUP, defined by the dividing lines on the appropriate figures) to whole-amylose AUP from wild type WXJ rice is larger than that from BEI & BEIIb knockout WXJ rice, while the ratio of AM2 AUP to whole-amylose AUP shows the opposite (Fig. 2a). The *X*_AM1_ and *X*_AM2_ values of both wild type WXJ rice and BEI & BEIIb knockout WXJ rice are similar (Table 1). The *X*_AM1_ ratio of Koshihikari rice is larger than that from Daohuaxiang rice, while the ratio of AM2 AUP shows the opposite. The AM2 peak of Daohuaxiang rice also is at a higher DP than that of Koshihikari rice (Fig. 2b). The *X*_AM1_ values of Daohuaxiang rice and Koshihikari rice are similar; however, the *X*_AM2_ value of Daohuaxiang rice is significantly higher than that of Koshihikari rice.

## Discussion

### Enzyme activity in amylopectin synthesis

There are several branching enzyme isoforms in rice grains, including BEI, BEIIa, BEIIb and BEIII (Mizuno et al. 1993). Our results shows that BEI and BEIIb knockout significantly reduces branching ability in rice endosperm: thus BEI and BEIIb knockout WXJ rice have significantly lower amylopectin content than wild type WXJ rice (Fig. 2a) (Zhu et al. 2012). While β_(i)_, β_(iii)_ and β_(iv)_ decrease significantly in the knockouts, the mutations have little effect on β_(ii)_ (Table 1). This result indicates that BEIIa is the main branching enzyme involved in the second enzyme set for amylopectin synthesis (15 < DP < 30), while BEI and BEIIb play important roles in the first, third and fourth enzyme sets. BEIIb is essential for the synthesis of short amylopectin chains (10 < DP < 15) and BEI is essential for synthesis of long amylopectin chains (DP > 30), a result consistent with previous work (Nakamura et al. 2010; Nakamura 2002). The absence of these two enzymes cannot be completely compensated by BEIIa, a result which has not been reported before. Knockout of BEI and BEIIb has more effect on the short amylopectin chains (essentially A and B_1_ chains) than on long ones (essentially B_2_ and B_3_ chains), as the *h*_(iii/i)_ and *h*_(v/i)_ values of BEI and BEIIb knockout WXJ rice are larger than those of the wild type. The differences between Daohuaxiang and Koshihikari amylopectin CLDs are not as large as those between wild type WXJ and BEI and BEIIb knockout WXJ rice (Fig. 1 and Table 1), which shows that the difference in enzyme sets in native rice lines is less than that between the wild type and this mutant. The differences in β values (Table 1) show that different rice lines have different activities for the SBE and SS enzyme sets. However, our results cannot separately distinguish the effects of SBE isoforms and SS isoforms, as the fitting only yields the ratios β of their activities.

The values of γ_(i,ii)_, γ_(iii,iv)_ and γ_(v,vi)_ of wild type WXJ and BEI and BEIIb knockout WXJ decreased in the mutant, although debranching enzyme (DBE) and SS remain unaffected. This is reflected in the BEI and BEIIb knockouts rice grains having less amylopectin: the debranching enzyme (mainly isoamylase, which controls the branch spacing to ensure that the CLD is crystallization-competent (Wu et al. 2013), and thus the amount of amylopectin (Fujita et al. 2003; Nakamura et al. 1997), works less efficiently. The values of γ_(i,ii)_, γ_(iii,iv)_ and γ_(v,vi)_ are similar between Daohuaxiang and Koshihikari rice, indicating DBE of those rice varieties work similarly. The γ values of the four different rice lines show that only the mutant line has a lower γ value, which might indicate that the predicted DBE function can be limited by the amount of its substrates.

The values of β_(iv)_ and β_(vi)_ of both WXJ samples are much lower than other β values, which indicates that enzyme sets iv and vi do not have much function in amylopectin synthesis in WXJ samples. However, the β values of Daohuaxiang and Koshihikari rice samples do not show much difference; all the enzyme sets play important roles in amylopectin synthesis.

### Enzyme activity in amylose biosynthesis

The ratio of the AM1 AUP of wild type WXJ is larger than that of BEI and BEIIb knockout WXJ; the shapes of the AM1 CLD are also different for the two samples, although they have similar *X*_AM1_ (Fig. 2). Debranched SEC results from previous studies have shown that the CLD of short amylose chains (AM1) also contains some long amylopectin chains, although the occurrence of band broadening complicates this conclusion (Horibata et al. 2004). BEI and BEIIb knockout WXJ has more long amylopectin chains than wild type WXJ, and thus AM1 of knockout WXJ should have more long amylopectin-chain contamination than that of wild type WXJ (Figs. 1, 2 and 3). This indicates that the AM1 AUP difference between knockout and wild type WXJ is not an artifact, and is not due to any long amylopectin chain contamination. The result could suggest that either or both BEI and BEIIb are involved in the synthesis of short amylose chains. *In vitro* experiments have shown that branching enzymes can catalyze the synthesis of amylose branches (Buleon et al. 1998; Takeda and Hizukuri 1987). BEI has a preference for transferring long amylopectin chains (Nakamura et al. 2010; Mizuno et al. 1993; Nakamura 2002). It could also have a role in transferring short amylose chains, which have been shown to have a similar CLD to those of long amylopectin chains in two-dimensional SEC weight distributions (Vilaplana and Gilbert 2010). Although the relative amount of long amylose chains increases in the mutant, the shape of the CLD of long amylose chains and *X*_AM2_ are similar for both samples, which shows that the molecular structure of long amylose chains remains unaffected. This indicates that BEI and BEIIb are probably not involved in the biosynthesis of long amylose chains, although other BEs must be involved to provide a chain-stopping mechanism. There seems to be an association between the higher β(i), β(iv), β(vi) and a lower *X*_AM2_ in Koshihikari. The lower β(i), β(iii), β(v) values are associated with the BEI and/or BEIIb mutant, which could imply that BEIIa is associated with β(ii), β(vi) and β(vi). Together, the above results suggest that BEIIa and/or BEIII might contribute to the shortening of long amylose chains. Further studies on other BE mutants and their amylose CLD could provide evidence for or against this inference.

The shapes of the amylose CLDs, ratios of AM1 AUP and of AM2 AUP and the *X*_AM2_ value of Daohuaxiang rice show clear differences from those of Koshihikari (Fig. 2b and Table 1). Both wild type WXJ and Koshihikari have higher β_(iv)_ (Table 1) and larger AUP of AM1 than the other two samples. This could indicate that besides GBSSI, enzymes responsible for synthesis of long amylopectin chains (such as the fourth enzyme set) might also play an important role in amylose synthesis. The samples with lower *h*_(v/i)_ value (wild-type WXJ and Koshihikari) have larger ratios of AM1 AUP, which also might indicate that the amount of long amylopectin and short amylose chains are negatively correlated. BEI is one of the enzymes that can affect synthesis of short amylose chains, as discussed above. The effect of SS is not analyzed in this study; however, SS involving long amylopectin chain synthesis might be able to affect short amylose chain synthesis. SSIII, which mainly contributes to the elongation of long inner amylopectin (B_2_, B_3_) chains (Edwards et al. 1999; Lloyd et al. 1999), could be another enzyme involved in synthesis of short amylose chains.

SSS (soluble starch synthase) is unlikely to be involved in the synthesis of long amylose chains, as there is nearly no amylose produced in GBSSI knockout grain (Myers et al. 2000; Denyer et al. 2001). The activity of GBSSI could therefore be responsible for the structural differences in long amylose chains of Daohuaxiang and Koshihikari. There is another possibility: that the AM2 CLD differences are because of pullulanase activity differences. The increased pullulanase activity would break down more uncrystallized amylopectin molecules and thus provide more sugar substrate for long amylose chain synthesis.

## Conclusion

Amylose chains in the wild-type and mutant rice samples examined here can be divided into two different groups, long and short, with different ranges of degrees of polymerization showing distinct features in the chain-length distribution. The present results show that these two amylose groups have different dominant biosynthesis enzymes. As is well known, long amylose chains are mainly synthesized by GBSSI. The synthesis of short amylose chains is found here to be controlled not only by GBSSI but also starch branching enzymes, which are also involved in long amylopectin chain synthesis. Because these smaller amylose chains have significant effects on functional properties such as mouth-feel and digestibility (Syahariza et al. 2013; Syahariza et al. 2014), this finding has potential application in the development of improved crop varieties.

## Methods

### Materials

Two groups of rice samples were used in this work. One group is the *japonica* cultivar Wuxiangjing 14 (WXJ) (17 % apparent amylose content) and its transgenic line with the BEI & BEIIb knockout (68 % apparent amylose content) (Zhu et al. 2012). The other group comprises two *japonica* rice cultivars with good cooking and eating quality, Daohuaxiang and Koshihikari. Both rice cultivars have same type of *Wx* allele (*Wx*^*b*^) with less amylose content (15 – 16 %) compared to rice lines with normal *Wx*^*a*^ allele. All rice lines were grown and harvested on the campus of Yangzhou University in 2012, and their mature seeds were ground into fine flour using a coffee grinder. Starch was extracted from rice flour and debranched using the method of Li et al. (2011). The debranched starch was freeze-dried overnight and stored in a desiccator prior to structural analysis.

### Fluorophore-assisted carbohydrate electrophoresis

The CLD of debranched amylopectin was analyzed using FACE, using an improved extraction method (Wu et al. 2014). Debranched starch (~0.3 mg) was incubated with 1.5 μL of a solution of 0.2 M APTS (8-amino-1,3,6-pyrene trisulfonic acid, 501309, Beckman-Coulter) in 15 % glacial acetic acid and 1.5 μL of 1 M sodium cyanoborohydride in tetrahydrofuran solution (296813, Sigma). The mixture was vortexed and incubated at 60 °C for 90 min for fluorophore labeling of the debranched amylopectin chains, following the method described in the Beckman-Coulter carbohydrate labeling and analysis kit. The solution of labeled glucans was diluted by adding 100 μL water. The mixture was centrifuged at 4000 *g* for 2 min and 70 μL of the supernatant was transferred to PCR tubes for immediate FACE analysis. The analysis was carried on a PA-800 Plus System using a solid-state laser-induced fluorescence detector with an argon-ion laser as the excitation source (Beckman-Coulter, Brea, CA, USA). A 50-μm diameter N-CHO coated capillary with separation length ~ 42 cm and carbohydrate separation buffer (both included in the carbohydrate labeling and analysis kit) were used for separating amylopectin branches. The sample was injected into the capillary by pressure injection for 3 s at 0.5 psi, and the separation of samples was driven with an applied voltage of 30 kV at 25 °C. The whole separation process takes 90 min. The areas of the peaks (which are essentially baseline-separated in FACE) directly correspond to the relative number glucan chains (the number CLD) in the peak.

### Fitting of amylopectin CLD using the biosynthesis model

The CLDs of amylopectin gained from FACE were fitted to a model developed by Wu, Gilbert and colleagues (Wu et al. 2013; Wu and Gilbert 2010), using a publicly available FORTRAN program package (Wu et al. 2013). The basic premise is that the CLD of chains spanning one, two and three lamellae is controlled by different enzyme sets, and that there are two sets per lamella, a total of six sets. The model has a number of parameters. Each enzyme set is parameterized by the ratio of activity of SBE to that of SS (β), that of debranching enzyme to that of SS (γ), the minimal chain length for an SBE to break an α-(1→4) linkage and attach the fragment on the same or on another chain to form a new branch (*X*_min_), and the minimum chain length for the portion remaining of the chain from which this is broken (*X*_0_). Each enzyme set in the six sets is mainly, but by no means exclusively, responsible for the synthesis of branches with a particular range of DP. The first and second enzyme sets are for A and B_1_ chains, the third and fourth enzyme sets are for B_2_ chains, and the fifth and sixth enzyme sets are for B_3_ chains. β_(i)_, β_(ii)_ etc. refer to the value of β for the first, second, etc., enzyme set. For a given plant species, there are two alternative models describing the interactions between two enzyme sets in a given lamella: the substrate-independent and substrate-competing models. For rice, it is found that the substrate-competing model is sufficient for quantitative fits (Wu et al. 2013). The additional parameters are then γ_(i,ii)_ and γ_(iii,iv)_, which are the sums of the two γ values of the first and second, and third and fourth, enzyme sets respectively. It is noted that, for reasons given in detail by Wu et al. (2010; 2013), the values of γ are not independent variables for a given enzyme set, but instead are fixed by a zero-eigenvalue relationship from the value of β, *X*_min_ and *X*_0_ for each enzyme set. The final parameters for the substrate-competing version of the model are *h*_(i,iii)_ and *h*_(i,v)_, which respectively are the ratios of the maximum of the CLD component synthesized by the enzyme set iii + iv combined to that of i + ii combined, and v + vi to that of i + ii.

Typical amylopectin CLDs show a number of features, including a global maximum in the range of the branches spanning one lamella, another peak or shoulder in the range spanning two lamellae and another in the range spanning three lamellae.

### Size exclusion chromatography analysis

The CLD of debranched starch was analyzed by SEC following the method of Li et al. (2011) Debranched starch (2 mg) was dissolved in DMSO with 0.5 % (w/w) LiBr for 2 h at 80 °C. This solution was analyzed with an Agilent 1100 Series SEC system (Agilent Technologies, Waldbronn, Germany) equipped with GRAM 100 and GRAM 1000 analytical columns (Polymer Standards Service (PSS), Mainz, Germany) set at 80 °C and a differential refractive index (DRI) detector (Wyatt, Santa Barbara, CA, USA). DMSO containing 0.5 % w/w LiBr was used as the eluent and the flow rate was 0.6 mL min^−1^. Calibration was with two pullulan standards of molecular weight 2.35 × 10^6^ and 1.22 × 10^6^; while the small range offered by these standards means that the conversion of elution volume to molecular weight (or DP), implemented by methods given elsewhere (Vilaplana and Gilbert 2010), will be inaccurate, use of even this limited calibration does mean that the data are reproducible. The results were collected and analyzed with Astra software (Wyatt) using a d*n*/d*c* value of 0.0717 mL g^−1^ (Li et al. 2011; Syahariza et al. 2013).

## Abbreviations

APTS: 8-Aminopyrene-1,3,6-trisulfonic acid, trisodium salt
SBE: Starch branching enzyme
CLD: Chain length distribution
DBE: Debranching enzyme
DP: Degree of polymerization
FACE: Fluorophore-assisted carbohydrate electrophoresis
GBSS: Granule-bound starch synthase
MOS: Malto-oligosaccharide
SEC: Size exclusion chromatography
SS: Starch synthase
WXJ: Wuxiangjing 14
